# Comatose within 6 weeks, but still alive after 3 years, Creutzfeldt‐Jakob disease with unusual progression

**DOI:** 10.1002/ccr3.2807

**Published:** 2020-04-19

**Authors:** Julia Kathleen Christopher, Brian Khong, Amin Abolfazli, Antonio Liu

**Affiliations:** ^1^ Department of Pulmonary and Critical Care Medicine UNLV Las Vegas Nevada; ^2^ Internal Medicine Adventist Health White Memorial Los Angeles California; ^3^ Ross School of Medicine North Brunswick New Jersey; ^4^ Neurology Adventist Health White Memorial Los Angeles California

**Keywords:** Creutzfeldt‐Jakob disease, neurodegenerative disease, rapidly progressive dementia

## Abstract

Creutzfeldt‐Jakob disease (CJD) should still be considered in a patient presenting with rapidly progressive dementia and negative CSF 14‐3‐3 protein and RT‐QulC. Treatable causes of encephalopathy must be ruled out. Neurodegenerative diseases must also be considered.

## Graphical abstract

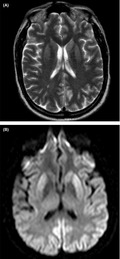

## 1 INTRODUCTION

Creutzfeldt‐Jakob disease, while rare, is the most common human prion disease (proteinaceous and infectious) which results in rapidly progressive dementia. It remains incurable with mortality typically within 12 months of diagnosis. Patients with CJD have a mutated form of PrP (PrP^Sc^). PrP^C^ is a normal, cell surface glycoprotein formed in the brain and guts with unknown normal function. PrP^Sc^ is synthesized in large quantities results in exponential misfolding and neurodegeneration. The proposed mechanism suggests the alpha helical regions of PrP^C^ bind to the beta pleated sheets of PrP^Sc^ and continue to propagate, similar to Alzheimer's disease and Parkinson's disease. Etiology for the mutated protein varies based on CJD type.^1^, ^2^

Creutzfeldt‐Jakob disease is the cause of 1/6000‐1/10 000 death per year in United States.

Three types of CJD exist^1^, ^2^:

1. Sporadic CJD (sCJD) (85%‐90%). It occurs without any identifiable pattern and appears to be a spontaneous mutation of PrP. Worldwide incidence is 1‐1.5 per 1 000 000 annually. The average age at death is 68 years old.
2. Genetic or familial CJD (5%‐15%). Two distinct forms are Gerstmann‐Straussler‐Scheinker syndrome and fatal familial insomnia. Both are more typical, rapidly progressive forms of the disease.
3. Infectious CJD includes dietary and iatrogenic (<1%). Dietary include two types: kuru and variant CJD. Kuru was found exclusively in a New Guinea tribe with supposed transmission via ritualistic cannibalistic practice.^2^ Variant CJD (vCJD), also known as “Mad Cow Disease,” was discovered in more than 200 people in Great Britain in 1994 who had eaten beef contaminated with the prion disease bovine spongiform encephalopathy. The disease manifested 10 years after an outbreak was observed in cattle.^2^ Moreover, iatrogenic cases of CJD have been recognized in patients who underwent cadaveric dura grafts, human growth hormone injections, corneal transplants, and procedures using contaminated neurosurgical instruments.^1^

Creutzfeldt‐Jakob disease may present with a prodrome of decreased concentration, memory loss, depression, and anxiety. As the disease progresses, affected patients may develop myoclonus, extrapyramidal symptoms, akinetic mutism, visual impairments, hypersomnia, psychiatric manifestation, and cerebellar disturbances. These patients progressively decline cognitively and functionally eventually becoming comatose then dying. Definitive diagnosis is by neuropathological, immunocytochemically, Western blot confirmed PrP^Sc^ or presence of scrapie‐associated fibrils. Probable diagnosis, after ruling out other etiologies, is by neuropsychiatric disorder, plus RT‐QulC analysis (99%‐100% specific) OR at least two of myoclonus, visual or cerebellar signs, pyramidal/extrapyramidal signs, akinetic mutism AND at least one of typical EEG, positive CSF 14‐3‐3 protein, or positive MRI.^1^ To date, there is no effective treatment; disease is uniformly fatal.

## 2 CASE REPORT

A 53‐year‐old male was brought to our Emergency Department with 1 month of confusion. He was completely normal 1 month prior, running a successful small business. The patient's wife initially observed confusion which worsened with impaired functioning, impairing his ability to work and care for his children. The patient was initially brought to an outside facility around 2 weeks into this illness. At that time, his symptoms had progressed to difficulty recognizing family, mutism, and hypersomnia. He then developed myoclonus with agitation and combativeness. MRI and CSF analysis from the outside facility were unremarkable (including an initially unavailable report of “unlikely prion disease” with no 14‐3‐3 protein detected). The patient was released with eventual admission to our hospital. By week 4 of his disease, he became bedridden, disoriented, and unable to recognize family. He developed myoclonic jerks and did not follow commands. Moreover, he had moderately increased muscle tone and incontinence. He never developed psychiatric manifestations. His clinical course continued to decline with subsequent intubation for airway protection by week 5 with deterioration by week 6 to GCS of 1/1/1. He has remained in this state ever since.

Past medical history was significant for remote history of nasal carcinoma. The patient took no home medications. His surgical history was significant for nasal carcinoma resection. Family history consisted of a father with hypertension. The patient denied using tobacco or drugs and used alcohol socially only. He ran a busy automobile mechanic business. He traveled to El Salvador 1 year prior to presentation. There are no recent sick or animal contacts.

**Figure 1:**
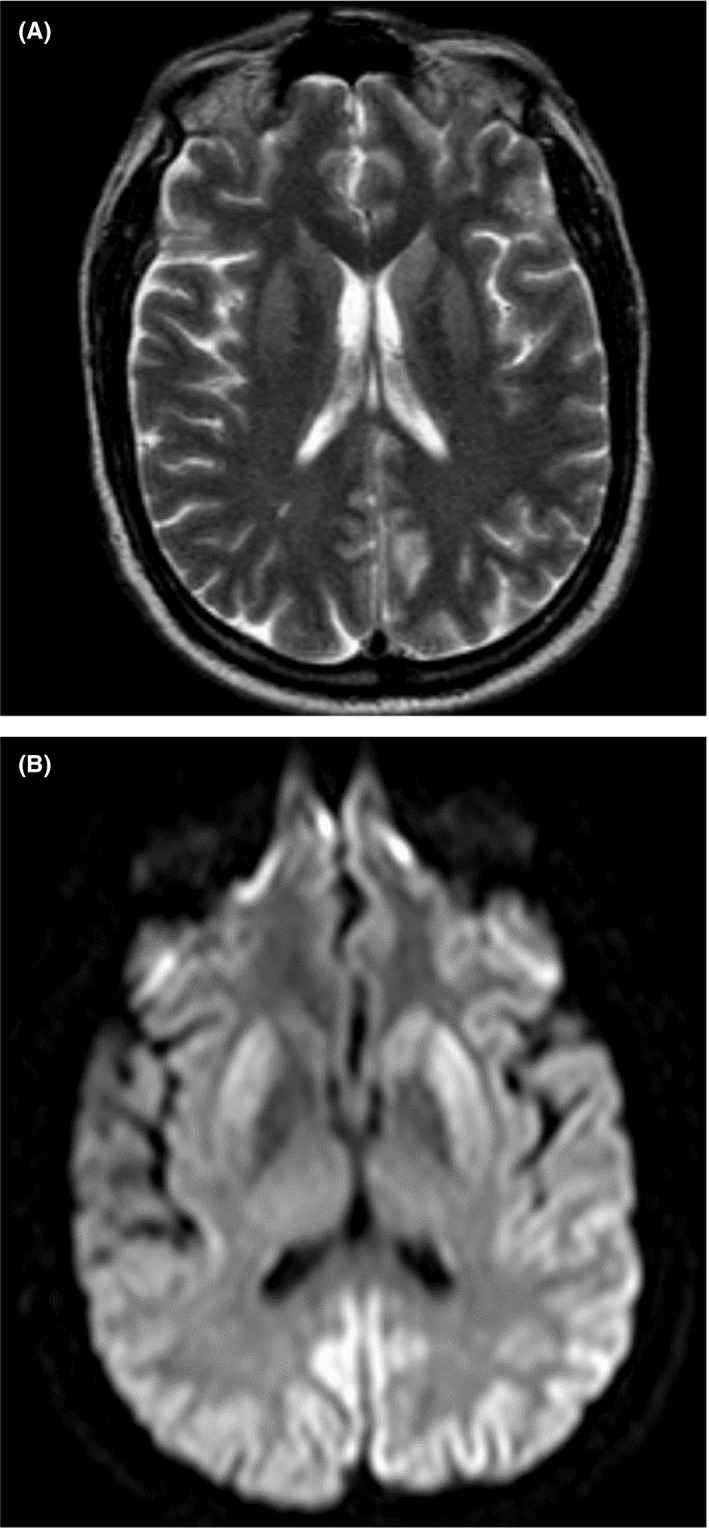
A, MRI abnormal signal density on bilateral caudate and basal ganglia on DWI. B, MRI abnormal signal density on bilateral caudate and basal ganglia on T2

**Figure 2:**
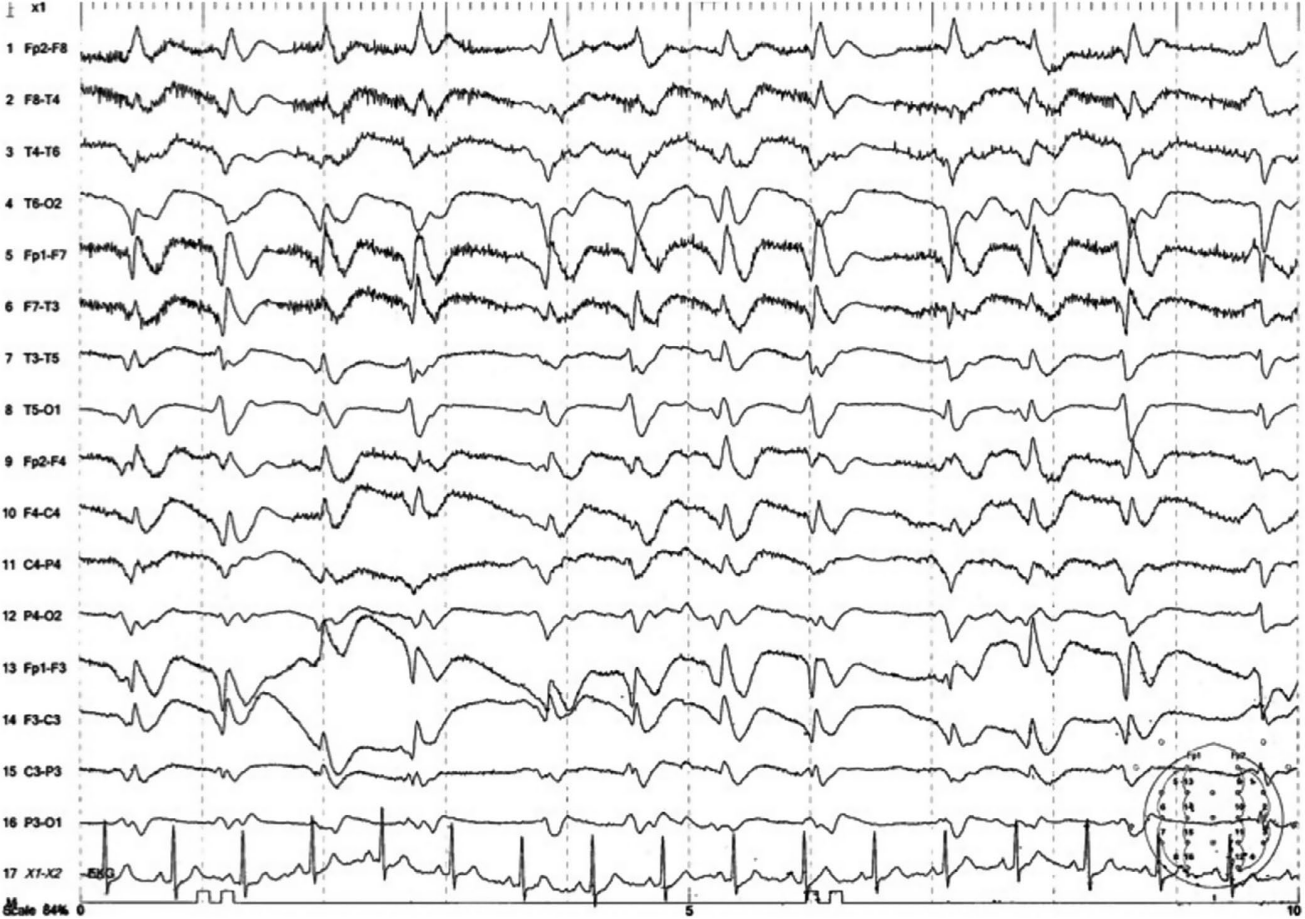
EEG showing periodic synchronized electric discharges on multiple EEGs refractory to seizure medication

At our hospital, he was never febrile with stable vital signs. After his initial negative MRI at the outside facility, MRI head began to show bilateral caudate and basal ganglia abnormality on T2 as well as DWI. Additionally, there is a cortical ribbon involvement in the posterior portion of both hemispheres (Figure 1A,B). There was never any pulvinar involvement. Routine complete blood count, complete metabolic panel, and coagulation studies were within normal limits. Serum analysis showed normal levels of vitamin B12, folate, methyl malonic acid, C‐reactive protein, erythrocyte sedimentation rate, Cryptococcus antigen screen, Treponema antibody EIA, and ceruloplasmin. N‐methyl D‐aspartate (NMDA) receptor antibodies and antineuronal antibody panel were negative. HIV was non‐reactive. EEGs showed periodic synchronized electric discharges which were refractory to antiepileptic drugs (Figure 2). Repeat lumbar puncture was performed and showed WBC 86/mm^3^ with 38% neutrophils, 34% lymphocytes, and 8% bands, RBC 3048 mm^3^, glucose level of 104 mg/dL, and protein level 100 mg/dL. CSF herpes simplex virus PCR, VDRL syphilis screen, West Nile IgM and IgG ELISA, and AFB stain were all negative. Cytology showed inflammatory cells but no malignant cells. Repeat CSF 14‐3‐3 protein and RT‐QuIC collected at 4 week after initial symptoms finally came back positive with probability of prion disease >98%. Brain biopsy, although requested, never took place.

After 2 months of acute hospitalization but continued stability, patient was placed at a nearby subacute facility. Follow‐up visits show no change nor deterioration. Patient remains on mechanical ventilation via tracheostomy and tube feeding at 3 year point from original symptoms.

## 3 DISCUSSION

Although CJD is a rare disease, it should be considered when a patient is presenting with a rapidly progressive dementia. Treatable causes of encephalopathy such as meningitis, meningoencephalitis, CNS vasculitis, hypothyroidism, sarcoidosis, toxicities, paraneoplastic syndromes, and primary neoplasms must be ruled out.^2^, ^3^ Neurodegenerative diseases such as Alzheimer's disease, dementia with Lewy bodies,^2^, ^3^, ^4^, ^5^ frontotemporal dementia, corticobasilar degeneration, and progressive supranuclear palsy must also be considered.^2^, ^3^ sCJD has even presented as a stroke.^5^

Considering rapidly progressive dementia, myoclonus, akinesia, mutism, positive CSF 14‐3‐3 protein and RT‐QulC, MRI high‐intensity signal abnormalities in the caudate nucleus on DWI and routine investigations that do not indicate an alternative diagnosis, the diagnosis of the patient is CJD, most probably the sporadic type.^8^

**Figure 3:**
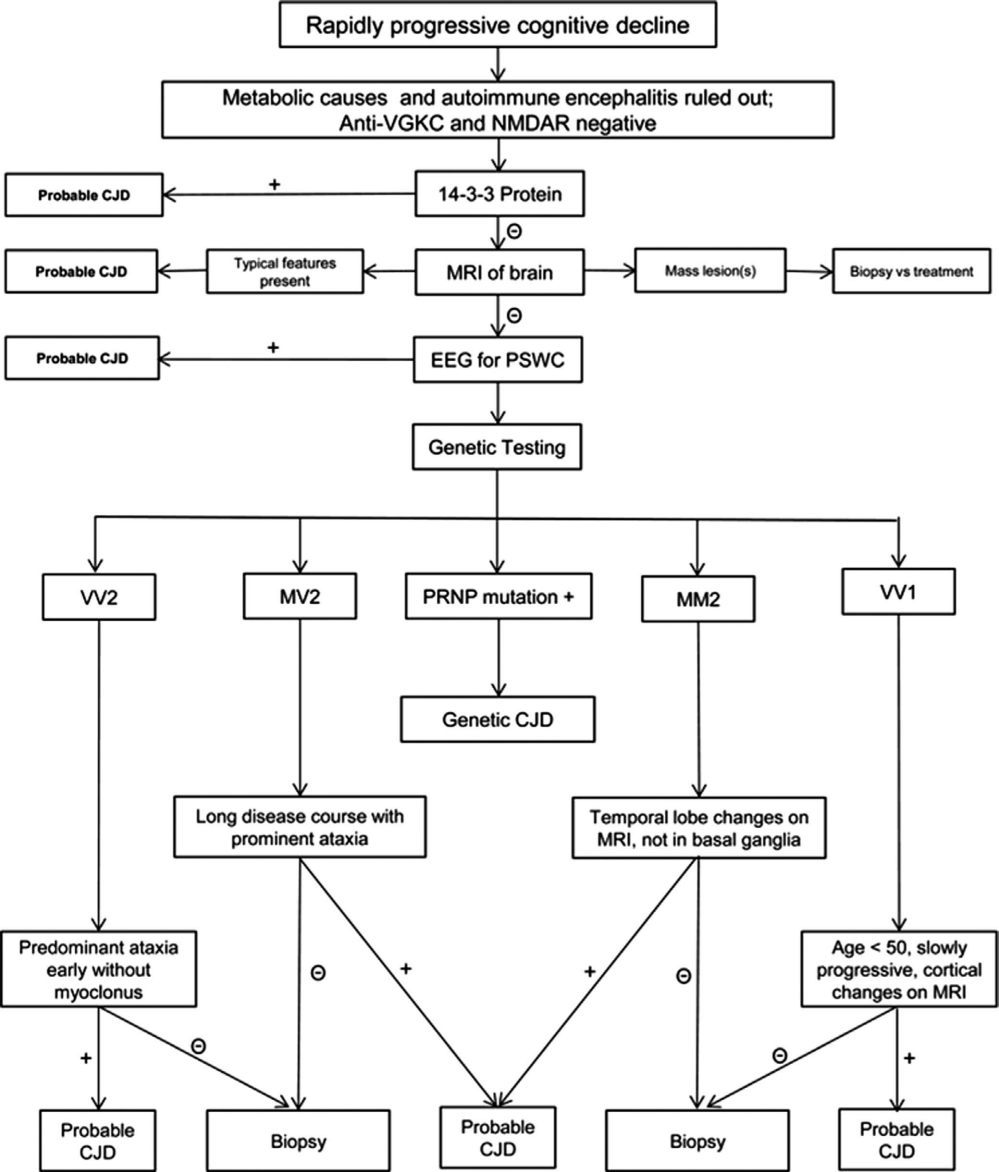
Proposed diagnostic pathway for the workup of a patient with rapidly progressive cognitive decline. NMDAR, N‐methyl‐D‐aspartate receptor; VGKC, voltage‐gated potassium channel (Source: https://thejns.org/focus/view/journals/neurosurgfocus/39/5/article‐pE2.xml or https://doi.org/10.3171/2015.8.FOCUS15328)

This patient case was remarkable in his progressive dementia given its uniquely significant rapidity over 6 weeks. Typically, onset of symptoms from presentation to a hospital ranges from 2 months to 2 years.^3^, ^4^, ^6^, ^7^ This patient's progression was so rapid that he became irreversibly comatose significantly prior to the turnaround time of CSF prion analysis. Furthermore, his CSF yielded no such protein when he was 2 weeks into his rapid deterioration. These discrepancies confounded the initial diagnosis. This case is also remarkable that the patient is still alive at the 3‐year point. sCJD has been documented to run a faster course than vCJD yet our patient does not have vCJD given no pulvinar sign and lack of psychiatric manifestations dominating the course of disease. Previous literature notes a patient with sCJD who had survived for 2 and a half years.^9^ Although the gold standard for diagnosis is tissue pathological confirmation, but brain biopsy was not performed at the early stages of diagnosis due to failure of securing neurosurgical service and facility, which is not uncommon at the community hospital settings. Later in the diagnosis process when CSF markers became positive, indication for brain biopsy was not justified considering risk‐benefit assessment, low sensitivity, and no change in treatment regardless of the result (Figure 3).^10^ Our patient is surviving beyond 3 years currently. He continues to remain unusual in his rapidity, development, and longevity.

## 4 CONCLUSIONS

Physicians should be aware that for a patient presenting with rapidly progressive dementia, Creutzfeldt‐Jakob Disease (CJD) should be included as one of the differential diagnoses. Since CJD can present with various forms of symptoms, and although a rare disease, it should not be ruled out by negative laboratory results and images. The gold standard diagnosis is confirmation by brain biopsy, but due to restrictions for operating room sterilizations, low sensitivity, high morbidity, and the fact that the positive result does not change the treatment in most cases, brain biopsy is not routinely done. It is very important to rule out treatable diseases in differential diagnosis such as meningitis, meningoencephalitis, CNS vasculitis, hypothyroidism, sarcoidosis, toxicities, paraneoplastic syndromes, and primary neoplasms. Our case depicts a case CJD in a fully functional patient who declined to a comatose state in 6 weeks. Although negative on initial workup, CSF 14‐3‐3 protein, and RT‐QulC became positive well over 6 weeks into his illness. This initial progression is unusually rapid. However, as of this article's submission date, the patient is still alive at a subacute facility 3 years after diagnosis. Both the rapid deterioration and protracted survival are atypical.

## CONFLICT OF INTEREST

None declared.

## AUTHOR CONTRIBUTIONS

AL: was the principal investigator and the attending neurologist, realized the presentation/time frame is abnormal, and initiated the write‐up. JC: was a resident treating the patient and working under Dr Liu, who did initial literature search and came up with the first draft. BK: was a resident working under Dr Liu, who contributed to the revision. AA: worked under Dr Liu's supervision on the final revision of the paper.
